# Clinical and radiological diagnosis of hypophysitis: a review of literature and own data

**DOI:** 10.14341/probl12777

**Published:** 2022-04-30

**Authors:** A. V. Vorontsov, D. M. Babaeva, V. P. Vladimirova, T. A. Dubovitskaya, A. O. Gavrilova, Zh. E. Belaya, N. G. Mokryshevа

**Affiliations:** Endocrinology Research Centre; Endocrinology Research Centre; Endocrinology Research Centre; Endocrinology Research Centre; Endocrinology Research Centre; Endocrinology Research Centre; Endocrinology Research Centre

**Keywords:** hypophisitis, magnetic resonance imaging, inflammatory disease of pituitary gland, lymphocytic inflammation

## Abstract

This article presents a literature review of the various forms of hypophysitis, its varieties, as well as the problem of radiation diagnosis and treatment of this pathology. Hypophysitis is a poorly understood and multifactorial disease which the difficulty of diagnosing is not only to a variety of nonspecific clinical manifestations and hormonal research data, but also the ambiguous results of MRI studies, the lack of clear MR patterns. The article reflects the main histological types of hypophysitis, the peculiarities of diagnosis in connection with general clinical symptoms, outlines the features of each type of hypophysitis with their own clinical observations. This review is devoted to modern ideas about the clinical course of hypophysitis, presented a set of characteristic diagnostic signs of the disease according to MRI and the treatment algorithms recommended today are also highlighted. The article summarizes data from foreign literature and our own clinical observations in order to develop an optimal protocol for MRI studies in patients with suspected hypophysitis, to develop recommendations for radiologists and endocrinologists for the correct results interpretation. The uniqueness of this review is the lack of data on the clinic, diagnosis and treatment of hypophysitis in the Russian literature today.

## Abstract (ru)

В настоящей статье представлен обзор литературы, посвященный особенностям клинического течения различных форм гипофизита, а также проблемам лучевой диагностики и лечения данной патологии. Гипофизит является малоизученным и многофакторным заболеванием, сложность диагностики которого обусловлена не только разнообразными неспецифическими клиническими проявлениями, данными гормонального исследования, но и неоднозначными результатами МРТ-исследования, отсутствием четких МР-паттернов. Данный обзор посвящен современным представлениям о клиническом течении гипофизитов, представлен набор характерных диагностических признаков заболевания по данным МРТ, также освещены рекомендуемые на сегодняшний день алгоритмы лечения. В статье обобщены данные зарубежной литературы и собственные клинические наблюдения с целью выработать оптимальный протокол МРТ-исследования у пациентов с подозрением на гипофизит, рекомендации для врачей-рентгенологов и эндокринологов для правильной интерпретации полученных результатов. Уникальностью данного обзора является отсутствие на сегодняшний день в российской литературе данных о клинической картине, диагностике и лечении гипофизита.

## INTRODUCTION

Hypophysitis is a rare inflammatory disease of pituitary gland characterised by a non-neoplastic infiltration into pituitary gland causing its dysfunction and swelling. Its prevalence rate is estimated at one new instance per 7–9 million population per annum. Hypophysitis accounts for 0.24% to 0.88% of all pituitary gland diseases [1].

Hypophysitis can be primary (idiopathic) or secondary. The former form of the disease is caused by an autoimmune inflammation in pituitary gland itself. The latter occurs as a result of systemic diseases or immunomodulator treatment [1]. Secondary hypophysitis may also be caused by cysts and tumours in sellar and suprasellar region, such as Rathke cleft cysts, craniopharyngiomas, adenoma, or germinoma [2–7]. Systemic diseases causing secondary hypophysitis include autoimmune disorders (Besnier-Boeck-Schaumann disease, haemochromatosis, Wegener’s disease, occlusive thromboaortopathy, Crohn’s disease, Langerhans cell histiocytosis, etc.) or infectious diseases (syphilis, tuberculosis, fungal and viral infections in immunocompromised patients) [8–12].

To date, five histological variants of primary hypophysitis have been reported: lymphocytic, granulomatous, xanthomatous, IgG4-related (plasmocytic), and necrotising ones. Moreover, mixed forms where two or more different histological variants of hypophysitis were simultaneously present have been reported [1–9][13].

Depending on the extent of inflammation across pituitary gland structure, hypophysitis is classified into several types: adenohypophysitis (anterior pituitary inflammation), infundibulohypophysitis (pituitary stalk and posterior pituitary inflammation), and panhypophysitis (inflammation across the entire pituitary gland). Adenohypophysitis occurs more frequently in females, whereas infundibulohypophysitis in males and children [1][4][7].

## Clinical presentation

Regardless of the cause of hypophysitis, typical features of its clinical presentation are related to pituitary gland swelling and include headache and sight disorders due to chiasmal compression. Depending on the extent of pituitary gland inflammation, hypophysitis involves pituitary gland dysfunction.

In 65% of cases, anterior pituitary is damaged, which causes partial or total hypopituitarism. In contrast to other forms of hypopituitarism, adrenocorticotropic hormone (ACTH) and thyroid-stimulating hormone (TSH) deficiency quite often occurs at early stages of primary hypophysitis; thus, such patients run a high risk of life-threatening adrenal insufficiency. The disease may also involve gonadotropin depletion, growth hormone deficiency or hyperprolactinemia. Less frequently (in about 10% of cases) infundibulohypophysitis occurs, which is due to posterior pituitary antidiuretic factor decrease as diabetes insipidus develops; sometimes, concomitant tropic hormone deficiency symptoms are observed.

In panhypophysitis, clinical manifestations of both anterior and posterior pituitary damage occur simultaneously; this type’s frequency is about 25% (see Table 1) [9].

**Table:** Table 1. Clinical presentation of  hypophysitis by volume of pituitary gland damage Note: adapted from [9].

Symptom, %	adenohypophysitis	infundibulohypophysitis	panhypophysitis
Headache	53	13	41
Vision disorders	23	3	18
Chronic adrenal insufficiency	42	8	19
Hypothyroidism	18	0	17
Hypogonadotropism	12	3	14
Hyperprolactinemia	23	5	17
Diabetes insipidus	1	98	83

## Primary hypophysitis

Lymphocytic hypophysitis

Lymphocytic inflammation is the most frequent histological variant of hypophysitis (68% of cases) [4]. It was first reported by Goudie and Pinkerton in 1962. This types occurs in females more often than in males, the proportion being 3 to 1 [3]; it develops most frequently in the fourth decade of life. In most cases, lymphocytic hypophysitis is diagnosed in the last month of pregnancy and in postnatal period, mainly during the first two postpartum months [3][12][14].

Granulomatous hypophysitis

Granulomatous hypophysitis was first reported in 1917 by Simmonds who examined 2,000 pituitary gland samples and found four cases of granulomatous inflammation not caused by tuberculosis of syphilis. This is an extremely rare disease (about 20% of all patients diagnosed with hypophysitis) that affects males and females in equal proportion. Granulomatous hypophysitis differs from lymphocytic one in that it is not associated with pregnancy, does not resolve spontaneously and does not occur simultaneously with other autoimmune diseases. Granulomatous hypophysitis has an unknown origin. In this type of hypophysitis, one has to rule out other diseases causing granuloma (such as tuberculosis, Besnier-Boeck-Schaumann disease or histiocytosis) before considering the pituitary gland granulomatous inflammation as primary [1][3][4].

Xanthomatous hypophysitis

Xanthomatous hypophysitis was first reported in 1998; it is less frequent than lymphocytic or granulomatous hypophysitis (about 3% of cases) [4]. It is believed that this variant may be triggered by an inflammatory reaction. It occurs more frequently in females (3 to 1 proportion), mostly in the fourth decade of life.

Necrotising hypophysitis

This is the rarest type of hypophysitis; it has been diagnosed in four patients aged 12, 20, 33 and 39. Three of these cases were histologically confirmed. It is mostly found in males [1][3].

IgG4-related hypophysitis

IgG4-related hypophysitis is often caused by systemic diseases, although may occur as a stand-alone one. It is more frequent in elderly males. Histological presentation is characterised by a dense lymphoplasmacytic infiltration of the affected pituitary gland tissue with prevalence of IgG4-positive plasma cells and fibrosis at advances stages of the disease [1–2][15]. Clinical presentation in IgG4-related hypophysitis is characterised by headaches, tunnel vision and galactorrhea. This disease often occurs with a concomitant damage to other body parts and systems (lymph glands, pancreas, hepatobiliary system, salivary glands, lacrimal glands, retroperitoneum, aorta, pericardium, thyroid gland, lungs, kidneys, skin, stomach, prostatic gland or ovaries).

Diagnosis of IgG4-related hypophysitis is confirmed by a typical histological presentation of a pituitary gland punctate. However, as pituitary gland biopsy is an invasive procedure, other criteria proposed in 2011 by Р. Leporati et al. may be used (see Table 2) [16][17].

**Table:** Table 2. Diagnostic criteria of IgG4-related hypophysitis Note: Diagnosis may be based on Criterion 1 or a combination of Criteria 2 and 3 or Criteria 2, 4 and 5.

Criterion 1	Histological confirmation.Lymphoplasmacytic infiltration of damaged tissue of pituitary gland with over 10 IgG4-positive plasma cells
Criterion 2	MRI confirmation.Pituitary gland and/or pituitary stalk swelling
Criterion 3	Histologically confirmed IgG4-associated disease in another organ
Criterion 4	Blood serum test confirmation.Elevated IgG4 in blood serum (over 140 mg/dl)
Criterion 5	Therapeutic response to glucocorticosteroids.Pituitary gland volume decrease and clinical improvement as a result of glucocorticosteroids treatment

## Secondary hypophysitis

Immunomodulator treatment induced

Immune checkpoint inhibitors are a new class of immuno-oncology drugs that is now widely used in cancer treatment. These drugs cause monoclonal antibodies to block CTLA 4 receptors (ipilimumab, tremelimumab) and PD 1 receptors (nivolumab, pembrolizumab) or produce an anti-PD1-L1 effect (avelumab, durvalumab, atezolizumab), thus reinforcing the T-cell antineoplastic immune response and achieving a positive therapeutic result in malignant tumor treatment [1–4]. However, interference with immune checkpoints may trigger an uncontrollable immune response thus causing an autoimmune damage of various organs, including pituitary gland via hypophysitis.

The time lag between the start of immunomodulator treatment and hypophysitis development will vary depending on the drug; some reports indicate that hypophysitis may occur several months after immune therapy initiation. The highest risk of hypophysitis is associated with ipilimumab treatment; this risk appears to be dose-sensitive: the frequency of hypophysitis is higher in patients receiving 10 mg/kg vs. those receiving 3 mg/kg. The risk of hypophysitis is lower in patients simultaneously receiving cytotoxic chemotherapy or brain radiotherapy, probably due to immune cells depletion. Clinical presentation is characterised by such non-specific symptoms as fatigue, headaches, myodynia, nausea and tropic hormone deficiency symptoms. Less frequent are chiasma syndrome and diabetes insipidus. Relatively rare symptoms such as mental confusion, hallucinations, unstable mood, anorexia, or shivers have been reported as well [1][13][17][18].

It is recommended that patients receiving immune checkpoint inhibitor treatment see an endocrinologist and regularly have hormone blood tests. About 90% of hypophysitis patients have ACTH deficiency; fatal acute adrenal insufficiency cases have been reported. Thus it is highly important to assess pituitary gland function prior to and during such treatment and to monitor the patients regularly. TSH deficiency is also frequent (over 80% of patients). It may be suggested that low TSH in blood serum is an early sign of hypophysitis induced by immune checkpoint inhibitors.

Patients suspected for hypophysitis are recommended to make an MRI scan of pituitary gland and see an ophthalmologist to conduct visual field testing on both eyes to rule out tunnel vision. In his study, Faje A. found that nearly 50% of hypophysitis patients and an MRI-detectable diffuse swelling of pituitary gland prior to any clinical symptoms [19][20].

It is important to note that high doses of glucocorticosteroids have no impact on the antineoplastic effect of immune checkpoint inhibitors and thus the treatment of patients with severe symptoms of hypophysitis and suprasellar compression syndrome should not be deferred. However, high doses of glucocorticosteroids do not alleviate ACTH deficiency, and most patients require long-term replacement therapy, when TSH and gonadotropin deficiency is often replenished at the same time. Whether such complication as hypophysitis is a reason to give up immuno-oncology drug treatment should be determined after considering all relevant factors in each case individually [1][3][4][7].

Hypophysitis associated with sellar and suprasellar tumors

Pituitary gland inflammation may be caused by suprasellar tumors. In such cases, inflammatory infiltrate is mostly lymphocytic or xanthogranulomatous and is focused near the lesion rather than distributed throughout the gland.

Germinoma is a rare brain tumor that occurs mostly in pre-puberty children. Isolated cases of secondary hypophysitis in children with germ-cell tumors have been reported in international literature [1].

Rupture of Rathke’s cleft cyst may cause hypophysitis manifesting as tunnel vision, headaches and hypopituitarism, very often including central diabetes insipidus. Some studies have found that many cases of xanthomatous hypophysitis may be in fact associated with rupture of Rathke’s cleft cyst [1].

Occurrences of secondary hypophysitis in combination with craniopharyngiomas or pituitary adenomas have been reported [1][7]. Our case is a female patient B. (35) that had excessive weight gain, high blood pressure and oedema. MRI scan showed a pituitary adenoma in combination with swollen pituitary stalk and uneven distribution of contrast medium therein.

## DIAGNOSTICS

## Magnetic resonance imaging

Pituitary gland biopsy followed by histological examination of the punctate is the golden standard of hypophysitis diagnostics. However, this method has a limited application as it is invasive. Thus, in patients for whom surgery is not indicated, hypophysitis is diagnosed based on clinical, laboratory and X-ray data. MRI is the first choice method when hypophysitis is suspected. Gutenberg A. еt al. have recommended using T2 weighted image (T2WI) and T1 weighted image (T1WI) modes on pre- and post-contrast coronal, sagittal and axial images (with slice thickness up to 3 mm) [8]. Diagnostic criteria of hypophysitis are the following: symmetrical pituitary gland enlargement (due to swelling) combined with a thickened pituitary stalk (sometimes, a normal size pituitary stalk is observed); diffuse inhomogeneity of signals from anterior pituitary and various degrees of cyst-like changes in anterior pituitary lobe structure; lack of typical hyperintensive MRI signal from posterior pituitary lobe on T1 weighted images. Changes in pituitary stalk will depend on the histomorphologic type of hypophysitis. From literature we learn that lymphocytic and granulomatous hypophysitis causes a diffuse damage across the entire pituitary gland; in these types of the disease, MRI scan images show a thickened pituitary stalk with inhomogeneous structure. In xanthomatous hypophysitis, changes may involve anterior pituitary lobe only, and thus MRI scan images will not show any changes in pituitary stalk. In some cases, changes in chiasma structure and visual tracts may occur (a hyperintensive MRI signal from chiasma and visual tracts on T2WIs). Data provided in the literature suggest that changes in chiasma and visual tracts may be observed in granulomatous (more frequently) and lymphocytic hypophysitis. Depending on the degree of inflammation in anterior pituitary, posterior pituitary lobe and pituitary stalk, an anatomic classification of hypophysitis has been made. According to it, hypophysitis is divided in three categories: adenohypophysitis, infundibulohypophysitis, and panhypophysitis (Figure 1). When conducting a MRI scan with intravenous contrast agent administration in active phase of the disease, intense and heterogeneous contrast agent absorption by anterior pituitary (in all types of hypophysitis) is observed. Contrast agent absorption by pituitary stalk will depend on the type of hypophysitis. Moreover, active contrast agent absorption by nearby pachymeninx thus forming a dura tail is observed [5][21].

**Figure:**
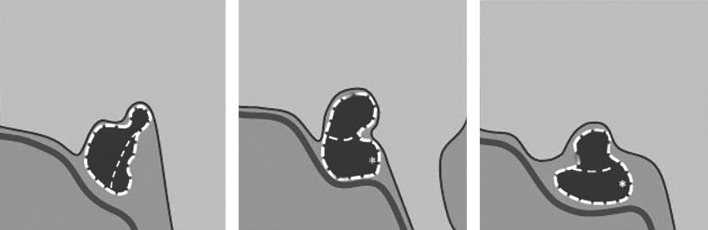
Figure 1. Schematic images of anatomic types of hypophysitisOn the left: adenohypophysitis with an enlarged anterior pituitary and unchanged pituitary stalk. In the middle: panhypophysitis with an enlarged anterior lobe of pituitary and pituitary stalk thickening and lack of the typical high signal from posterior pituitary lobe on T1WIs. On the right: infundibulohypophysitis with a lack of the typical strong signal from posterior pituitary on T1WIs, pituitary stalk thickening and a triangular-shaped pituitary gland deformation (Chiloiro S. et al. Department of Endocrinology, School of Medicine, Catholic University of the Sacred Heart).

When hypophysitis is suspected, MRI scan results also enable conducting differential diagnosis and ruling out pituitary adenoma (for all age categories), germinoma and Langerhans cell histiocytosis in adolescent patients, craniopharyngioma, Rathke’s cleft cyst and other tumors in suprasellar region and secondary changes in hypothalamus-pituitary region combined with hypopituitarism and diabetes insipidus [4][5].

Our evaluation of pituitary gland size is based on our experience, which suggests that in 99% of healthy individuals, pituitary gland vertical dimension is within 8 or 8.5 mm. In our view, this size can be considered the upper boundary of the norm [5]. Sagittal and transverse dimensions are most often determined by the size of sella turcica. Pituitary stalk thickness varies; most often, it is within 1–3 mm with possible deviations in distal and proximal parts.

Diagnostic criteria of hypophysitis may also include findings of patient monitoring. During treatment (with corticosteroids), a relatively rapid pituitary gland and pituitary stalk shrinking is observed (sometimes it is temporary, followed by a relapse in the form of pituitary gland swelling). At advanced stages of the disease, atrophy of anterior pituitary, empty sella turcica syndrome and fibrosis changes in anterior pituitary structure may occur. Lack of hyperintensive signal from posterior pituitary on T1WIs often persists through the advanced stage as well. Long-term monitoring reveals changes in MRI scan images taken with contrast agent administration: subtotal or total reduction of contrast agent absorption by anterior pituitary lobe, all the way to presence of contrast-free areas which must be due to fibrosis loci in anterior pituitary lobe structure [4][5][22].

In 2009, an attempt was made to objectivise and systematise diagnostic criteria of hypophysitis based on MRI scan findings in order to achieve better precision in differential diagnosis of hypophysitis and pituitary adenoma so as to avoid unwarranted surgical treatment. Eventually, 8 key predictors enabling a differential diagnosis were identified. They included patient’s age; pregnancy in females; pituitary gland size; character and speed of contrast agent absorption; symmetric or asymmetric pituitary gland swelling; posterior pituitary visualisation; pituitary stalk changes; changes in mucosa at sphenoid bone sinuses and/or nearby cribriform cells. A scoring system for MRI scan findings was proposed. This approach did not gain traction [5].

In our Centre, we are also considering the plethora of characteristic hypophysitis signs (described above) and believe that MRI scan findings are legitimate grounds to diagnose hypophysitis, provided that clinical and laboratory data are taken on board.

Hypophysitis should be suspected in patients with pituitary gland dysfunction and/or neurological (headaches) or ophthalmological (tunnel vision) anomalies if MRI scan images show typical signs of pituitary gland damage. Hypophysitis is usually diagnosed in acute stage when pituitary gland swelling and pituitary stalk thickening occur due to inflammation infiltrate and disease symptoms and signs are manifested. As a chronic phase evolves, it is characterised by irreversible hypopituitarism due to pituitary gland fibrosis and subsequent empty sella turcica syndrome [4][5] (Figures 2–5).

**Figure:**
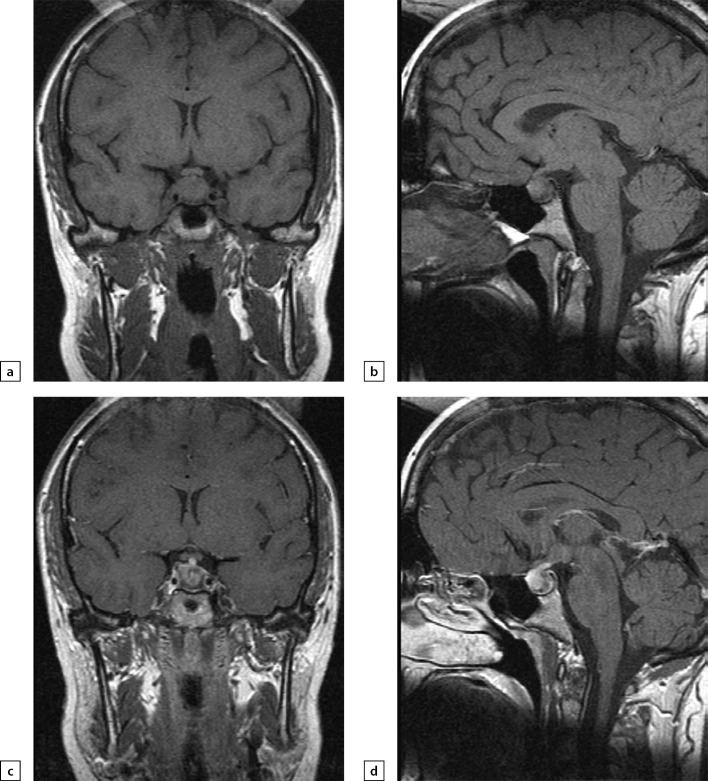
Figure 2. Female patien K (36)Lymphocytic hypophysitis. First MRI scan made in 2014 due to galactorrhea and headache: a) T1WI, coronal slice; b) T1WI, sagittal slice; c) T1WI, post-contrast coronal slice; d) T1WI, post-contrast sagittal slice. The scan found pituitary gland swelling and inhomogeneous structure, pituitary stalk thickening and diffuse/inhomogeneous contrast agent absorption.

**Figure:**
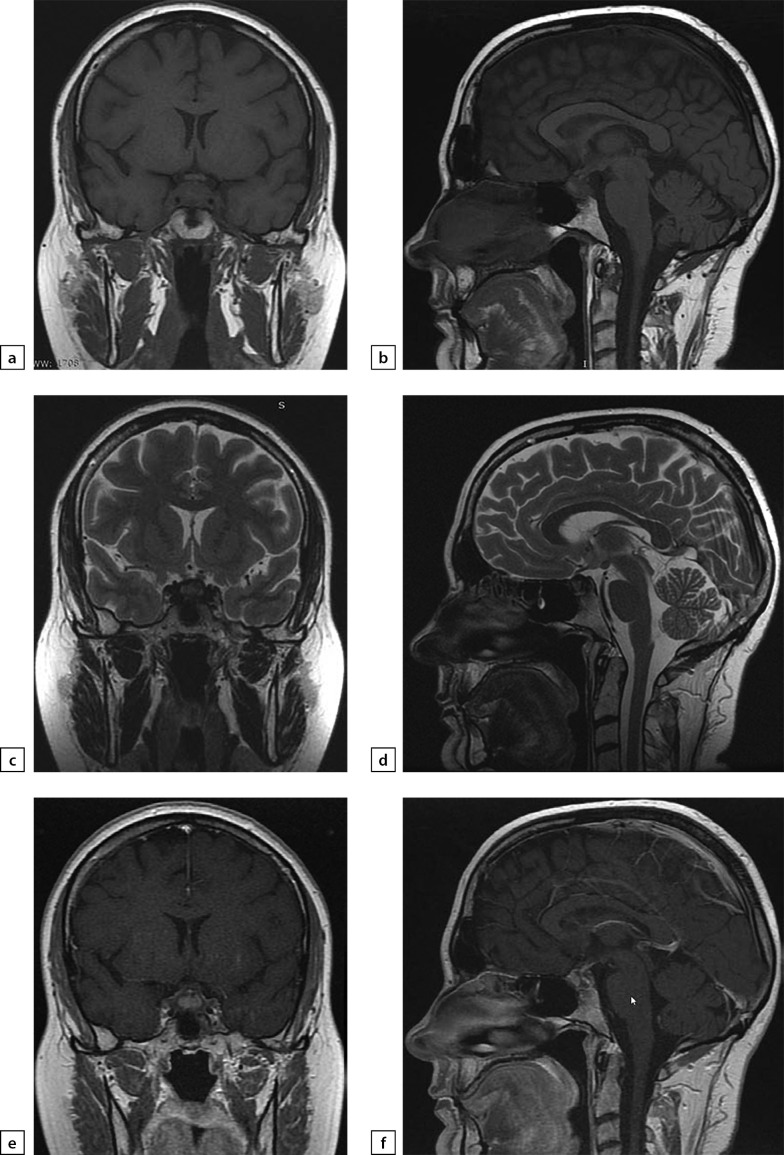
Figure 3. The same patient as above2019 MRI scan images, made after histological verification: a) T1WI, coronal slice; b) T1WI, sagittal slice; c) T2WI, coronal slice; d) T2WI, sagittal slice; e) T1WI, post-contrast coronal slice; f) T1WI, post-contrast sagittal slice. The scan found more visible pituitary gland swelling and inhomogeneous structure, including due to appearance of fibrous changes and chiasmal compression.

**Figure:**
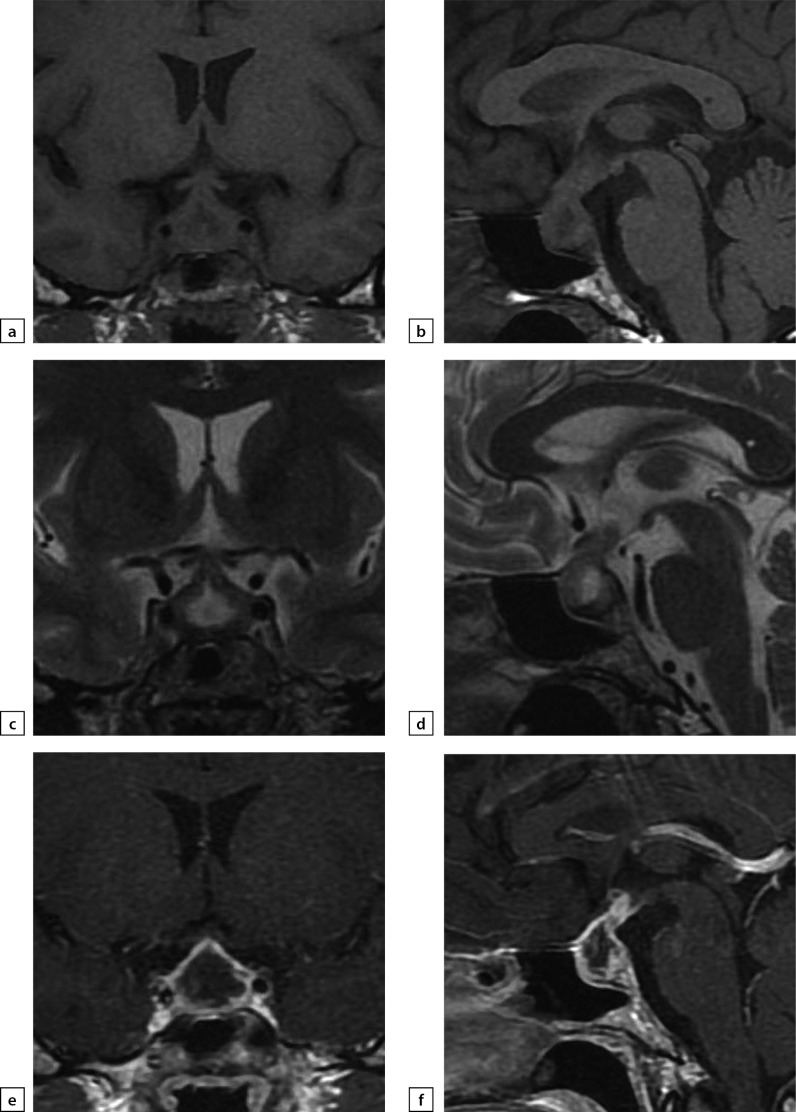
Figure 4. Female patient E (38)Lymphocytic hypophysitis. Substantial headaches, menstrual disorder, face numbness on the right-hand side. A MRI scan was conducted on 24 June 2019: a) T1WI, coronal slice; b) T1WI, sagittal slice; c) T2WI, coronal slice; d) T2WI, sagittal slice; e) T1WI, post-contrast coronal slice; f) T1WI, post-contrast sagittal slice. The scan found pituitary gland deformation with bulging upper end and inhomogeneous structure. At the centre, a liquid component with dimensions 5×7×5.5 mm was identified (infundibulohypophysitis). At pituitary gland periphery, intensive contrasting was observed. Pituitary stalk is deformed and evenly thickened to 4.5 mm; the stalk absorbs contrast agent intensively and unevenly. The typical signal from posterior pituitary is not identified. Suprasellar cistern is narrowed.

**Figure:**
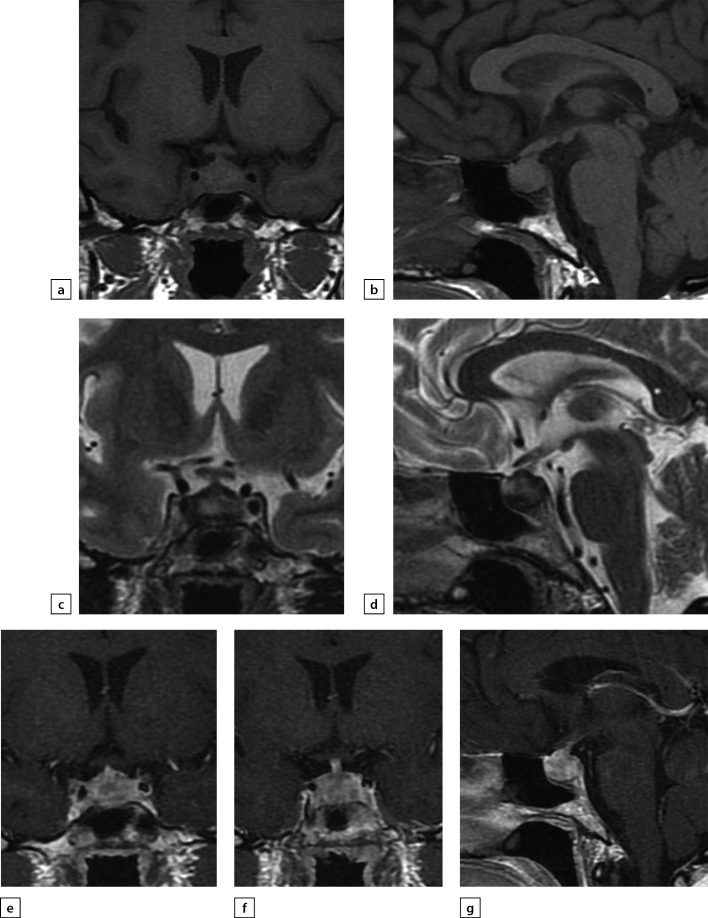
Figure 5. The same patient as aboveFollow-up MRI scan images taken on 24 September 2019 while large doses of glucocorticosteroids were administered: a) T1WI, coronal slice; b) T1WI, sagittal slice; c) T2WI, coronal slice; d) T2WI, sagittal slice; e) T1WI, post-contrast coronal slice; f) T1WI, post-contrast coronal slice; g) T1WI, post-contrast sagittal slice. The scan found pituitary gland swelling and deformation with bulging upper end and inhomogeneous structure. With contrast agent administration, an intense and uneven contrast agent absorption is observed. Pituitary stalk is deformed and thickened primarily in distal part (up to 3.5 mm); the stalk absorbs contrast agent evenly and intensively. The typical signal from posterior pituitary is not identified. Suprasellar cistern is narrowed; distance to chiasm is 1 mm. Active contrast agent absorption by sella turcica diaphragm is observed. Pituitary gland dimensions have decreased versus the previous MRI scan taken on 24 June 2019.

## Laboratory diagnostics

Autoimmune etiology of primary hypophysitis occurs due to presence of antibodies to pituitary gland; the following antigens are considered targets of such autoimmune aggression: α-enolase, growth hormone, hypophysitis-specific factors 1a and 2 (PGSF1a and PGSF2), pituitary gland-produced regulator ferments (PC1/3, PC2, CPE, and 7B2), secretogranin II, Chromosome 14 open reading frame (C14orf166), specific transcription factor TPIT and chorionic somatomammotropin (HCS). In order to identify antibodies to pituitary gland in primary hypophysitis, we used several methods (enzyme multiplied immunoassay), radioligand binding analysis, immunoblotting and immunofluorescence); the frequency of antibody-positive hypophysitis is 11% to 73% depending on the tested antigen(s) and the method used. However, the role of such antibodies remains unclear; neither are they specific to hypophysitis. Thus, antibodies to pituitary gland were identified through indirect immunofluorescence in about 45% of patients with hypophysitis confirmed histologically, but they were also found in blood serum in patients with isolated central diabetes insipidus (35%), germinomas (33%), deficiency of anterior pituitary-produced tropic hormones (29%), prolactinomas (27%), Rathke’s cleft cysts (25%), craniopharyngiomas (17%), non-functioning pituitary tumours (13%), somatotropinoma (12%), and in healthy individuals (5%). They may also be found in patients having other autoimmune disorders, especially in chronic autoimmune thyroiditis [23][24].

## Clinical case

Male patient Z (56) was hospitalised in 2017 due to substantial headaches, skin dryness, weight loss, and lack of appetite. Tests revealed pan-hypopituitarism: secondary hypothyroidism (TSH at 0.32 mIU (0.4–4.2); free thyroxine at 4.3 pmol/L (7.86–14.4)); secondary hypogonadism (follicle stimulating hormone at 1.81/L (1.6–9.7); luteinising hormone at 0.591/L (2.5–11); testosterone at 0.773 nmol/L (11–33.5)); secondary hypocorticism (cortisone in the mornings at 100.5 nmol/L (123–626); adrenocorticotrophic hormone at 22.51 pg/mL (7–66)), and diabetes insipidus. A brain MRI scan was conducted that found infiltrative damage to pituitary gland that is typical for hypophysitis (Figure 6). As pan-hypopituitarism treatment, replacement therapy with sodium levothyroxine, desmopressin and glucocorticosteroids was prescribed.

**Figure:**
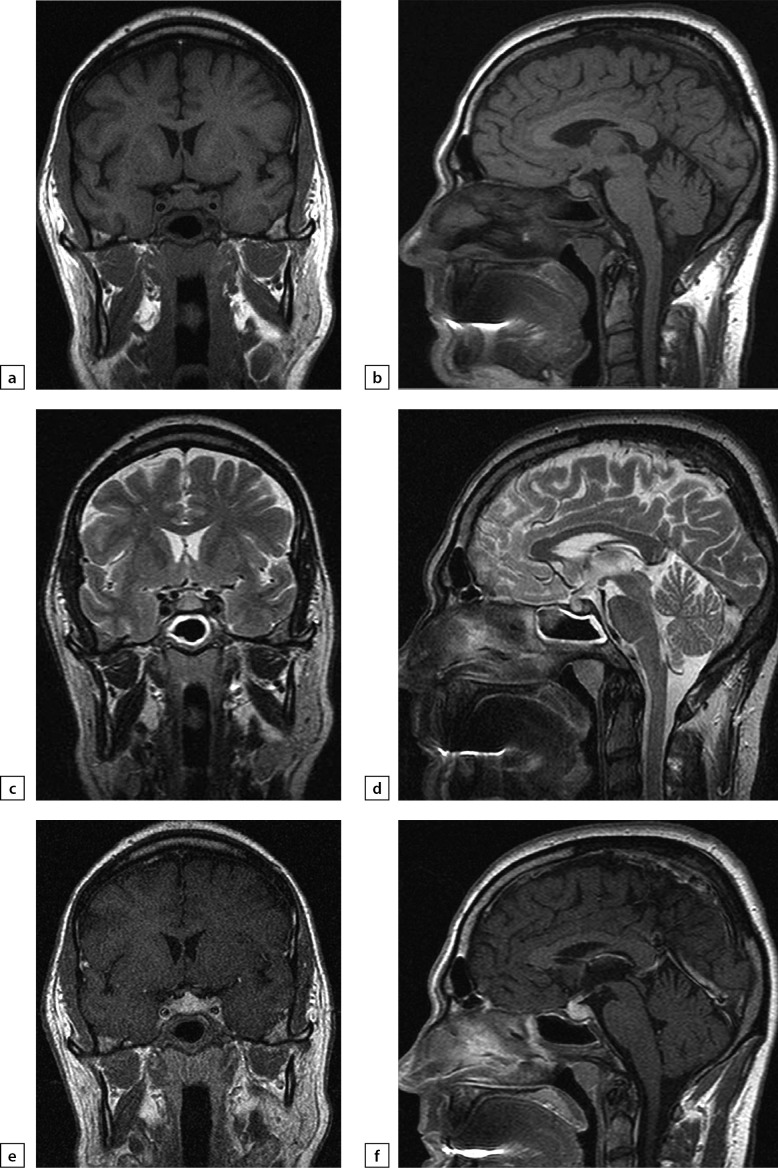
Figure 6a) T1WI, coronal slice; b) T1WI, sagittal slice; c) T2WI, coronal slice; d) T2WI, sagittal slice; e) T1WI, post-contrast coronal slice; f) T1WI, post-contrast sagittal slice. The scan found pituitary gland swelling and deformation. Pituitary stalk is displaced forwards and is 3.5 mm thick. With contrast agent administration, a visible uneven contrast agent absorption across pituitary gland is observed.

In order to differentiate the diagnosis from germinoma, a blood test for β-subunit of human chorionic gonadotropin (β-HCG) and α-fetoprotein was recommended. This test was carried out on 21 June 2017: β-HCG was measured at under 2 ng/mL (norm: under 2.0), and α-fetoprotein at 1.87 IU/mL (norm: under 7.29). Histiocytosis was ruled out based on lungs and skull CT findings.

At hospital, the patient underwent pulse therapy with methylprednisolone for hypophysitis; in total, 12 g of methylprednisolone was administered. After the treatment, brain MRI scan revealed a decrease of pituitary gland dimensions against the previous image; pituitary stalk thickness decreased as well (Figure 7). The patient reported a better condition due to less severe headaches.

**Figure:**
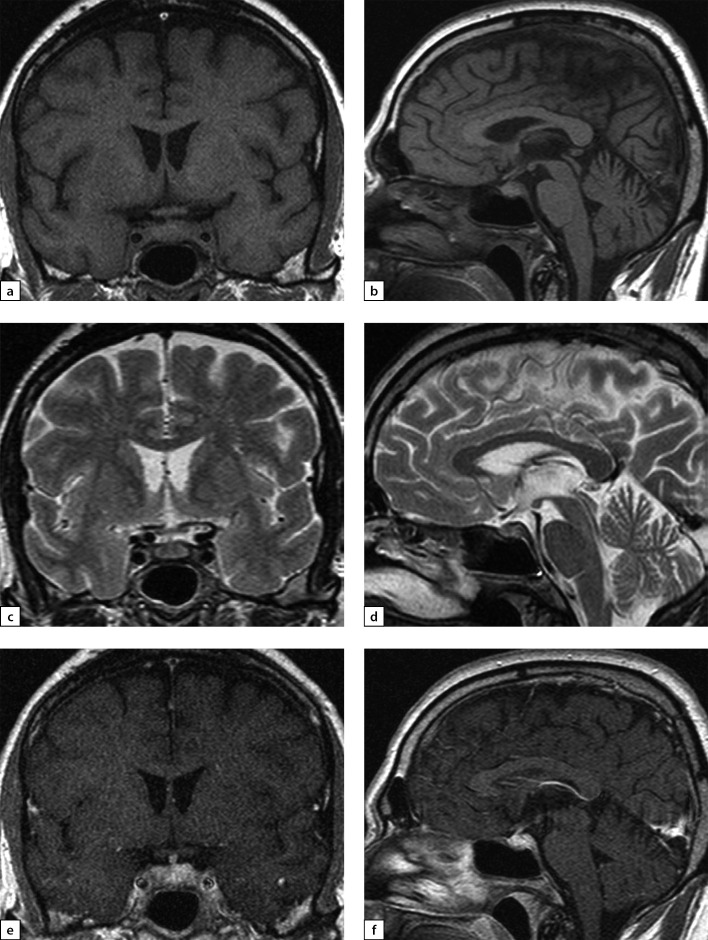
Figure 7a) T1WI, coronal slice; b) T1WI, sagittal slice; c) T2WI, coronal slice; d) T2WI, sagittal slice; e) T1WI, post-contrast coronal slice; f) T1WI, post-contrast sagittal slice. The scan found a decrease of pituitary gland’s vertical dimension and a change in its structure.

However, two weeks later, a relapse of cephalgic syndrome occurred. The patient started self-administered treatment with dexamethasone (2–4 mg/day); a positive effect was observed. On 17 November 2017, transnasal transsphenoidal hypophysectomy with endoscopic assistance was conducted due to suspected hypophysitis. Histological analysis confirmed lymphocytic hypophysitis. The test found fragment of anterior pituitary with areas of atrophy, fibrosis, and focal lymphoplasmacytic stromal infiltrations mixed with neutrophils and eosinophils. Separately, two smaller fragments of vitreous connective tissue with severe lymphoid infiltration and three small fragments of anterior pituitary with signs of atrophy, ­fibrosis and severe lymphoplasmacytic stromal infiltrations were identified.

Following the surgical treatment, a brain MRI scan found a post-hypophysectomy condition with empty sella turcica (Figure 8).

**Figure:**
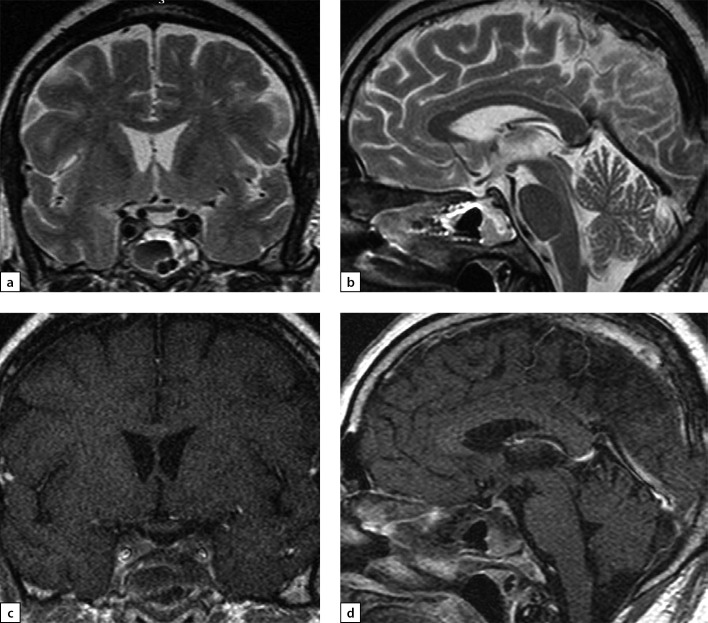
Figure 8a) T2WI, coronal slice; b) T2WI, sagittal slice; c) T1WI, post-contrast coronal slice; d) T1WI, post-contrast sagittal slice. Suprasellar cistern is prolapsing into sella turcica. Post-surgery changes manifest as a flattened and deformed pituitary gland; pituitary stalk is not thickened. The typical signal from posterior pituitary is not identified.

In April 2018, a relapse of severe headaches occurred. At the time, the patient was receiving replacement therapy: sodium levothyroxine 100 μg/day and desmopressin 30 μg/day in the evenings (irregularly due to headaches). In addition, the patient continued to take intramuscular injections of 2 mg dexamethasone once every 1–2 days. An ophthalmologist found neuropathy of the right-side trochlear nerve. At hospital, dexamethasone was replaced with methylprednisolone 8 mg/day and thus cephalgic syndrome was once again relieved. A brain MRI scan was made which found additional tissue in the right-side cavernous sinus and cerebellar tentorium thickening on the right-hand side (an adverse development); thus, an expansive pachymeninx process was taking place (Figure 9).

**Figure:**
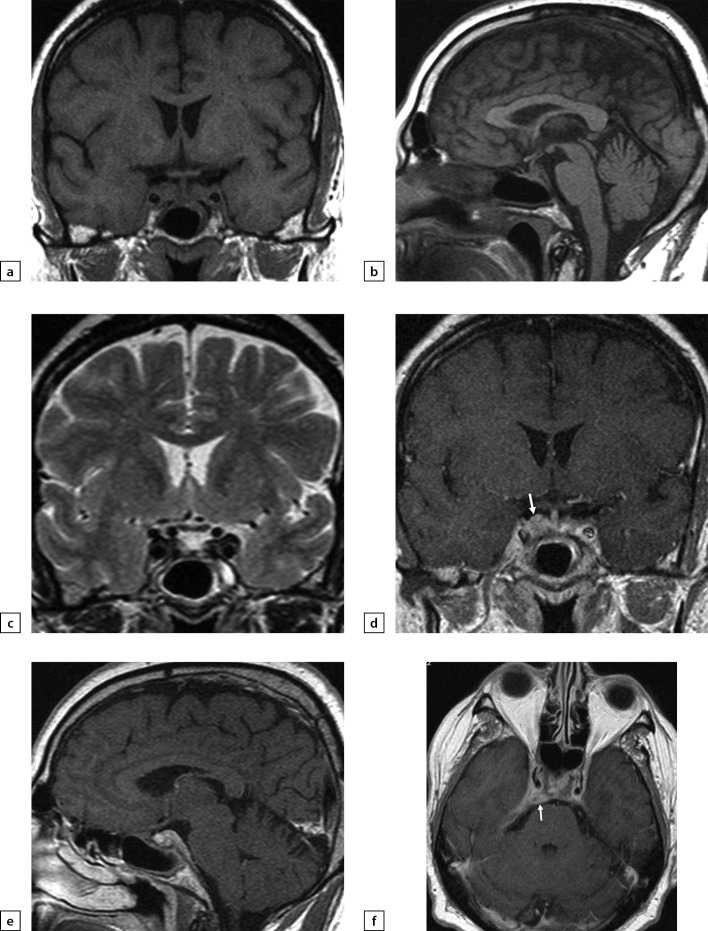
Figure 9a) T1WI, coronal slice; b) T1WI, sagittal slice; c) T2WI, coronal slice; d) T1WI, post-contrast coronal slice; e) T1WI, post-contrast sagittal slice; f) T1WI, post-contrast axial slice. Post-surgery changes in pituitary gland structure were observed. Thus, in the right-side cavernous sinus and in the sphenoid bone wing region, an additional tissue was found. With contrast agent administration, it actively absorbed contrast agent. A connection between this mass and a thickened cerebellar tentorium (also actively absorbing contrast agent) and interpeduncular cistern coating was identified (see arrow).

Due to secondary hypogonadism, on 25 July 2018, following a measurement of prostate-specific antigen level (0.2 ng/mL), an injection of 1000 mg testosterone undecanoate was administered. In September and December 2018, the second and third testosterone undecanoate injections were administered. A follow-up MRI scan made 6 months later revealed positive developments: a decrease of the amount of additional tissue in the right-side cavernous sinus (Figure 10).

**Figure:**
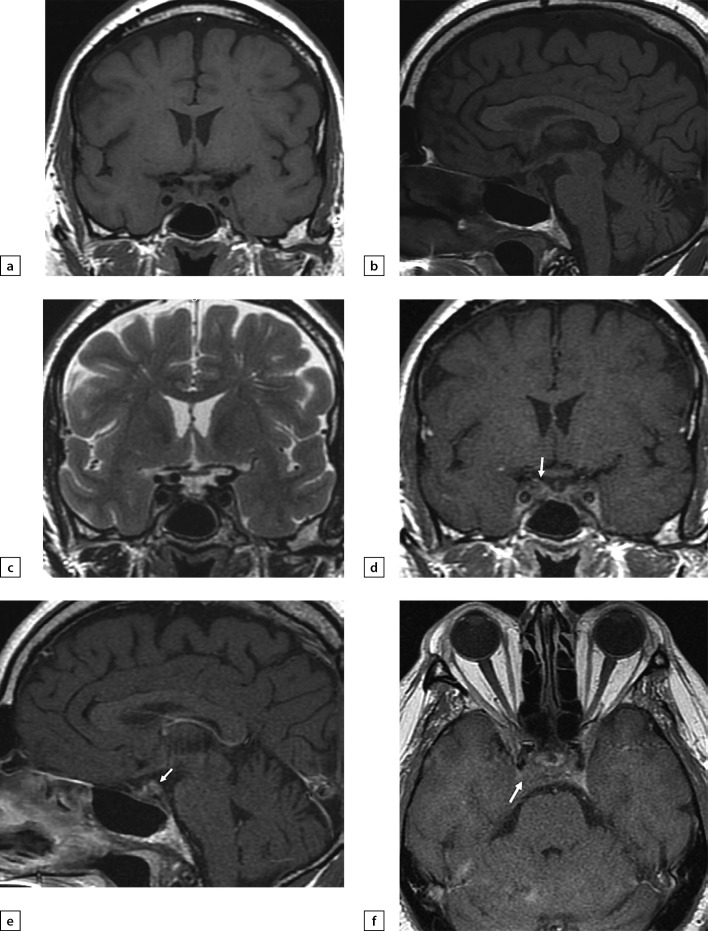
Figure 10a) T1WI, coronal slice; b) T1WI, sagittal slice; c) T2WI, coronal slice; d) T1WI, post-contrast coronal slice; e) T1WI, post-contrast sagittal slice; f) T1WI, post-contrast axial slice. A pachymeninx thickening in the region of right-side cavernous sinus and in the sphenoid bone wing region was observed. No pathological absorption of contrast agent took place (see arrow).

This clinical case underscores the fact that any surgical treatment should be done based on solid evidence.

Following the treatment, Patient Z is feeling well and has no major complaints. The patient is receiving replacement therapy for pan-hypopituitarism. Follow-up MRI scans reveal no further changes. An ophthalmologist could not confirm neuropathy presence or absence. No disorders affecting peripheral visual fields were identified.

## DIFFERENTIAL DIAGNOSTICS

First and foremost, it should be noted that correct interpretation of structural changes in chiasmosellar region requires analysis of clinical presentation and findings of hormonal and x-ray tests. Thus, germinomas appearing in suprasellar region are difficult to differentiate from hypophysitis in children due to their similar clinical characteristics (diabetes insipidus + premature sexual development + tunnel vision). A correct diagnosis is important since these two diseases require completely different treatment and entail completely different outcomes. Biopsy-confirmed primary hypophysitis occurs very rarely in children and adolescents; thus, in children under 10 germinoma should be considered the more likely diagnosis. Tumour markers, such as α-fetoprotein, β-subunit of human chorionic gonadotropin or placenta-like alkaline phosphatase in cerebrospinal fluid may be valuable criteria to diagnose germinoma. However, pituitary gland biopsy remains the golden standard of the relevant diagnostics.

It ought to be noted that hypopituitarism, which is a common manifestation in hypophysitis and germinoma, may appear long before germinoma can be visible on MRI scan images.

Correct diagnosis differentiating between hypophysitis and pituitary adenoma or Sheehan syndrome in postnatal female patients is necessary.

In patients with adenoma, a hormonal blood test may find gonadotropin, TSH or ACTH deficiency similar to that observable in patients with hypophysitis; however, it should be noted that adrenal deficiency is the earliest sign of hypophysitis. It is followed by secondary hypothyroidism and hypogonadotropic hypogonadism; a reduced somatotrophic hormone (STH) level occurs rarely. Some authors have suggested conducting tests with dopamine agonists to differentiate hypophysitis from prolactinoma. In the latter case, the tumour size will decrease, whereas no such effect will be observed in hypophysitis patients. Pituitary adenoma will often manifest on MRI scan images as an asymmetric pituitary gland enlargement, sometimes with a displacement of pituitary stalk with no changes therein. These effects may in rare cases evolve to include a cavernous sinus. The typical T1WI signal from posterior pituitary will be always present. With contrasting agent administration, pituitary adenoma is characterised with low-scale, low-speed contrast agent absorption, whereas the rest of anterior pituitary remains unchanged [1][5][8].

A feature common for hypophysitis and Sheehan syndrome is that both will often occur postpartum. Sheehan syndrome is an ischemic necrosis and a persistent hypofunction of pituitary gland due to postnatal haemorrhage. This complication of delivery causes a substantial blood loss and is also refered to as postnatal hypopituitarism [5][17]. The main difference between hypophysitis and Sheehan syndrome on MRI scan images is that that in the latter case, pituitary gland enlargement takes place simultaneously with haemorrhagic changes; an oedema of visual tract or chiasma may also occur [5][18]. First symptoms of hypophysitis will typically manifest at later stages of pregnancy, whereas Sheehan syndrome develops postpartum. Moreover, Sheehan syndrome will prevent postpartum lactation, which occurs very seldom in lymphocytic hypophysitis. An empty sella turcica syndrome may eventually be observed on MRI scan images in Sheehan syndrome [12, 14].

In general, differentiating between hypophysitis and these other diseases may be difficult due to their similar manifestations. Diagnostics must involve a MRI scan with intravenous contrast agent administration for all patients suspected for hypophysitis (Figure 11) [13][14].

**Figure:**
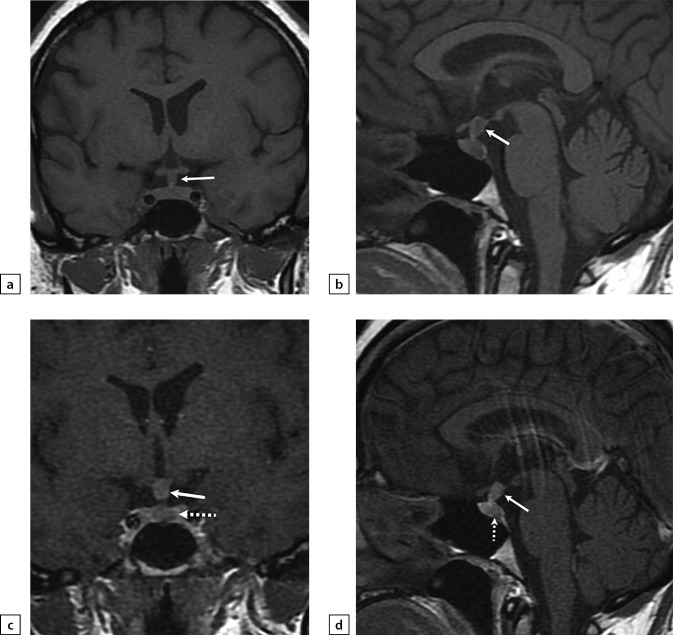
Figure 11. Female patient B (35)The patient complained on headaches, weight gain, blurred vision and thirst. A secondary hypophysitis combined with an ACTH-producing pituitary adenoma. An MRI scan was made: a) T1WI, coronal slice; b) T1WI, sagittal slice; c) T1WI, post-contrast coronal slice; d) T1WI, post-contrast sagittal slice. The scan found pituitary stalk thickening (see arrow). With contrast agent administration, an inhomogeneous contrast agent absorption by pituitary stalk and presence of adenoma in anterior pituitary structure (see dotted arrow) were observed.

## TREATMENT

Hypophysitis treatment should aim at alleviating the inflammation and countering pituitary gland enlargement and at compensating the hormonal deficiency arising from pituitary gland dysfunction.

For immunosuppressive therapy, glucocorticosteroids and cytostatic drugs are used. Immunosuppressive therapy should be initiated in acute stage of hypophysitis only, in order to alleviate the inflammation and counter pituitary gland enlargement and thus, to prevent the evolution of clinical symptoms. Glucocorticosteroids are considered first-line drugs for immunosuppressive therapy [1][3][4][25].

At present, there are no protocols for hypophysitis patients’ management. There are neither any straightforward recommendations as to glucocorticosteroids doses or treatment duration.

Positive effect of immunosuppressive therapy with glucocorticosteroids is described in 15% to 41% of primary hypophysitis cases and in 42% to 64% of cases of hypophysitis caused by immunomodulator treatment.

Cases of restoration of gonadotropin, TSH and STH emission and restoration of posterior pituitary lobe function due to immunosuppressive therapy with glucocorticosteroids have been reported. However, immunosuppressive therapy with glucocorticosteroids may be followed by relapse of the disease in up to 38% of cases.

In the event of resistance against glucocorticosteroids and emergence of signs of an exogenous hypercorticoidism, cytostatic drugs such as azathioprine, methotrexate and cyclosporine may be effective. However, assessing a deferred therapeutic effect may be difficult [10][11].

For patients with secondary hypothyreosis, replacement therapy should be initiated only following the correction of hypocorticism; otherwise, addisonian crisis may occur as adrenal deficiency is not compensated [11]. As to hypogonadotropic hypogonadism and STH deficiency, replacement therapy is not recommended at the acute stage of the disease; it may be postponed to the chronic stage of the disease following immunosuppressive therapy with glucocorticosteroids [13]. For patients with hypophysitis caused by immunomodulator treatment, replacement therapy for hypogonadism should be indicated depending on the neoplasia type; drugs with recombinant human growth hormone are counter-indicative [1][12][25].

Surgical treatment can be indicated in cases when tunnel vision, blurred vision, cranial palsy or lack of positive therapeutic effect of drug treatment occur. Surgical treatment may remove pituitary gland compression against chiasmosellar region; however, patient follow-up has revealed a relapse of the disease in 25% of patients within three years. Post-surgery, pituitary gland function was restored in 8% of patients only; smoothing of chiasmal syndrome is also observed quite rarely [25][26].

Stereotactic radiation therapy (X-ray surgery) proved effective for treatment of several patients with relapse of lymphocytic hypophysitis [1][23].

## PATIENT FOLLOW-UP

It is recommended that pituitary function and replacement therapy adequacy be evaluated every month in patients at acute stage of hypophysitis. A repeat MRI scan is recommended three months following the initial diagnosis and every six months thereafter. In hypophysitis arising from immunomodulator treatment with neurological symptoms, monthly pituitary gland MRI scans are recommended. At acute stage of hypophysitis, pituitary gland MRI scans should be carried out whenever any relapse symptoms occur [11].

## CONCLUSION

At present, there are no universally accepted protocols for hypophysitis diagnostics and management. Even though hypophysitis is identified nowadays more frequently than before, making a correct diagnosis remains a complicated task. Diagnosing a hypophysitis is difficult as many other diseases manifest similar clinical presentation and similar hormonal disorders; moreover, there is no univocal X-ray pattern for hypophysitis. Diagnosing a hypophysitis can be made easier if follow-up hormonal and X-ray tests are conducted; such follow-up tests will make the diagnosis more explicit for both clinicians and radiologists. We recommend conducting brain MRI scans with contrast agent administration for all patients having a cephalgic syndrome that cannot be alleviated with nonsteroidal antiinflammatory drugs and having hormonal disorders; such scans should be made as early as possible, since many types of hypophysitis exist and follow-up scans may show changes in pituitary gland structure. This study illustrates the main radiological types of hypophysitis and reports a clinical case of a patient with headaches that could not be alleviated with drugs. Based on our data, we recommend conducting a repeat brain MRI scan with contrast agent administration three months following the first scan for all patients with suspected hypophysitis. Modern-day methods of hypophysitis diagnostics will enable avoiding unwarranted surgical treatment.

## ADDITIONAL INFORMATION

Funding source. This study was conducted on the authors’ own accord. No funding was raised.

Conflict of interest. The authors hereby declare no actual or potential conflict of interest related to this publication.

Authors’ contribution. Alexander V. Vorontsov: research design and concept, literature analysis, drafting this article and approval of the final version of the manuscript; Diana M. Babaeva: research design and concept, literature analysis, drafting this article and approval of the final version of the manuscript; Victoria P. Vladimirova: research design and concept, literature analysis, drafting this article and approval of the final version of the manuscript; Tatiana A. Dubovitskaya: research design and concept, literature analysis, drafting this article and approval of the final version of the manuscript; Alina O. Gavrilova: research design and concept, literature analysis, editing the text, drafting this article and approval of the final version of the manuscript; Zhanna E. Belaya: editing the text and approval of the final version of the manuscript; Natalia G. Mokrysheva: editing the text and approval of the final version of the manuscript. Every author approved the final version of the text prior to publication and agreed to accept responsibility for all aspects of this study, which implies due investigation and resolution of any issue related to the accuracy or integrity of any part thereof.

Patient’s consent. Informed consent to publication of depersonalised personal medical data (in this journal specifically) has been voluntarily provided by patients.
